# Plasmonics Meets Metasurfaces: A Vision for Next Generation Planar Optical Systems

**DOI:** 10.3390/mi17010119

**Published:** 2026-01-16

**Authors:** Muhammad A. Butt

**Affiliations:** Institute of Microelectronics and Optoelectronics, Warsaw University of Technology, Koszykowa 75, 00-662 Warsaw, Poland; ali.butt@pw.edu.pl

**Keywords:** plasmonics, metasurfaces, hybrid plasmonic system, modulators, nanolaser

## Abstract

Plasmonics and metasurfaces (MSs) have emerged as two of the most influential platforms for manipulating light at the nanoscale, each offering complementary strengths that challenge the limits of conventional optical design. Plasmonics enables extreme subwavelength field confinement, ultrafast light–matter interaction, and strong optical nonlinearities, while MSs provide versatile and compact control over phase, amplitude, polarization, and dispersion through planar, nanostructured interfaces. Recent advances in materials, nanofabrication, and device engineering are increasingly enabling these technologies to be combined within unified planar and hybrid optical platforms. This review surveys the physical principles, material strategies, and device architectures that underpin plasmonic, MS, and hybrid plasmonic–dielectric systems, with an emphasis on interface-mediated optical functionality rather than long-range guided-wave propagation. Key developments in modulators, detectors, nanolasers, metalenses, beam steering devices, and programmable optical surfaces are discussed, highlighting how hybrid designs can leverage strong field localization alongside low-loss wavefront control. System-level challenges including optical loss, thermal management, dispersion engineering, and large-area fabrication are critically examined. Looking forward, plasmonic and MS technologies are poised to define a new generation of flat, multifunctional, and programmable optical systems. Applications spanning imaging, sensing, communications, augmented and virtual reality, and optical information processing illustrate the transformative potential of these platforms. By consolidating recent progress and outlining future directions, this review provides a coherent perspective on how plasmonics and MSs are reshaping the design space of next-generation planar optical hardware.

## 1. Introduction

Modern optical systems are increasingly constrained by the size, complexity, and rigidity of conventional bulk components [1]. As emerging applications such as immersive displays [2,3], compact imaging [4,5], biomedical sensing [6,7], high-speed communication [8,9], and optical information processing [10] continue to expand, there is a growing demand for optical hardware that is thinner, lighter, faster, and more functionally versatile than traditional lens and waveguide-based architectures [11]. These demands have driven intense interest in planar and nanoscale approaches that enable optical functionality to be implemented directly at material interfaces rather than accumulated through long propagation paths [12,13].

Plasmonics [14,15] and metasurfaces (MSs) [16,17,18,19] have independently matured into powerful platforms for nanoscale light manipulation. Plasmonic structures exploit collective electron oscillations at metal–dielectric interfaces to confine optical fields far below the diffraction limit, enabling ultrafast light–matter interaction, strong optical nonlinearities, and extreme miniaturization of active devices [20,21]. MSs, in contrast, consist of planar arrays of subwavelength scatterers that enable precise control over optical phase, amplitude, polarization, and dispersion within layers only hundreds of nanometers thick [11,22]. Through appropriate design of their constituent meta-atoms, MSs can replace bulky refractive or diffractive elements with flat optical components capable of wavefront shaping, beam steering, holography, and imaging [23,24,25,26,27,28].

While plasmonics and MSs rely on distinct physical mechanisms, their functional roles are increasingly complementary. Plasmonic elements provide strong field localization and high-speed modulation over nanometer-scale interaction volumes, making them well-suited for modulators [24], detectors [25], nanolasers [29], and nonlinear optical elements [12]. MSs, on the other hand, excel at spatially distributed wavefront control and multifunctional optical processing, enabling complex transformations of optical fields in free space or in hybrid environments [26,27,28]. When combined within hybrid plasmonic–dielectric or plasmonic–MS architectures, these strengths can be leveraged simultaneously, allowing systems to achieve performance levels unattainable by either platform alone [30,31].

Recent progress in materials science and nanofabrication has further accelerated this trend. Alternative plasmonic materials, including transparent conducting oxides, transition metal nitrides, and two-dimensional materials, offer improved tunability, reduced loss in selected spectral regimes, and compatibility with large-scale fabrication [21,32,33]. In parallel, advances in MS design methodologies and manufacturing techniques such as deep-ultraviolet lithography, nanoimprint lithography, and multilayer stacking are enabling large-area, high-yield production of complex planar optical components [34,35]. These developments have made it increasingly feasible to co-integrate plasmonic and MS functionalities within unified planar platforms.

This review surveys the fundamental principles, material platforms, and device-level implementations of plasmonic, MS, and hybrid plasmonic–dielectric systems, with a particular emphasis on interface-mediated optical functionality. Section 2 and Section 3 revisit the physical foundations of plasmonics and MSs, highlighting design strategies and performance trade-offs relevant to planar and hybrid architectures. Subsequent sections examine representative devices and applications, including ultracompact modulators and detectors, metalenses, beam steering systems, programmable optical surfaces, and emerging optical information processing schemes. Key challenges related to optical loss, dispersion, thermal management, and large-area fabrication are critically discussed, followed by a forward-looking perspective on how plasmonics and MSs are shaping the future of flat, multifunctional, and programmable optical systems.

## 2. Rethinking Plasmonic Fundamentals for Planar and Interface-Engineered Optical Systems

Plasmonics is based on the excitation of surface plasmon polaritons and localized surface plasmon resonances at metal dielectric interfaces, enabling electromagnetic confinement far below the diffraction limit [36]. While the physical foundations of these modes are well-established, their relevance is increasingly defined by planar optical architectures in which functionality is concentrated at engineered interfaces rather than distributed along extended propagation paths. In this setting, plasmonic loss and limited propagation length are no longer dominant constraints but are outweighed by the advantages of extreme field localization, ultrafast response, and strong light-matter interaction [37,38].

Surface plasmon polaritons originate from collective electron oscillations coupled to electromagnetic waves at metal dielectric boundaries and exhibit dispersion relations that deviate strongly from the free-space light line. This behavior enables precise control of modal confinement, group velocity, and the local density of optical states, allowing plasmonic structures to support slow light effects, enhanced spontaneous emission, and tailored mode coupling within compact footprints [39]. For planar systems that prioritize localized interaction and high functional density, these dispersion characteristics provide a powerful mechanism for engineering optical response at surfaces and interfaces rather than long-distance waveguides.

Localized surface plasmon resonances supported by metallic nanostructures such as nanorods, nanodisks, and bowtie antennas further expand the available design space by concentrating optical fields into nanoscale hotspots [40,41]. These localized fields enhance nonlinear optical processes, amplify Raman scattering, and strongly modify radiative emission pathways. Recent advances in inverse electromagnetic design allow systematic tailoring of resonance spectral position, symmetry, and near-field distribution, enabling efficient coupling to gain media, molecular emitters, and quantum systems [42,43]. As feature sizes and inter-element gaps approach the subnanometer regime, quantum mechanical effects such as electron tunneling and nonlocal response become increasingly significant, marking the onset of quantum plasmonic behavior [44,45].

Material selection remains a key factor in determining plasmonic performance and functionality [46,47]. Noble metals such as gold and silver continue to serve as reference materials, but are limited by interband absorption, chemical instability, and challenges in large-scale fabrication [48,49]. Transition metal nitrides, particularly titanium nitride, offer improved thermal stability and compatibility with complementary metal oxide semiconductor processes [50]. Aluminum enables plasmonic operation in the ultraviolet and visible spectral regions [51], while copper provides a cost-effective alternative when oxidation can be effectively managed [52]. Transparent conducting oxides, such as indium tin oxide, introduce epsilon near-zero regimes in which strong field enhancement and ultrafast tunability can be achieved [53]. Two-dimensional materials, including graphene, support highly confined and electrically tunable plasmons in the mid-infrared, enabling dynamically reconfigurable plasmonic responses at planar interfaces [54].

The functional role of plasmonic building blocks is also evolving. Plasmonic waveguides are increasingly valued not for long-distance transport but for maximizing interaction strength within minimal physical length [55]. Antennas are no longer passive scattering elements but multifunctional components capable of coupling, concentrating, modulating, and reradiating optical fields within engineered environments [56]. Resonant plasmonic structures provide sharp spectral selectivity and enhanced field confinement, enabling compact filtering, sensing, and switching functionalities. New coupling strategies between plasmonic elements and dielectric or MS-based structures enable controlled energy exchange across different confinement regimes without relying on extended propagation [36].

Together, these developments highlight a shift in how plasmonic physics is interpreted within modern optical systems. Rather than serving as a lossy alternative to conventional guiding, plasmonics is increasingly recognized as an enabling platform for interface-mediated optical functionality in planar and hybrid architectures. By localizing electromagnetic energy precisely where optical processing occurs at surfaces, boundaries, and engineered nanostructures, plasmonic modes provide a foundation for compact, ultrafast, and highly responsive optical components that naturally complement MSs and other planar photonic platforms [57].

## 3. MS Fundamentals Through a Forward-Looking Lens

MSs have redefined the concept of wavefront engineering by demonstrating that optical phase, amplitude, and polarization can be controlled within a planar layer of subwavelength scatterers [26,58]. Generalized Snell’s law captures the essence of this approach by showing that abrupt phase shifts at an interface can redirect light in ways unattainable by conventional optics [59]. As MSs move from laboratory demonstrations to real integrated devices, the emphasis is shifting from simple beam steering toward complex, multifunctional responses that can operate across broad bandwidths and variable incident conditions [60].

The meta-atom is the primary building block of MSs [61]. Dielectric meta-atoms based on Mie resonances can produce strong electric and magnetic responses with minimal absorption loss [62]. This dual response allows designers to achieve full two pi phase control while maintaining high transmission efficiency. Metallic meta-atoms, while more lossy, continue to be valuable for achieving polarization rotation, broadband absorption, and tunable behavior [28]. The future of MS engineering likely lies in hybrid meta-atoms that combine the strengths of dielectric confinement and plasmonic field intensification. Such structures can open new parameter spaces for multifunctional devices that simultaneously control polarization, spectral response, and wavefront curvature [30,63,64].

Functional control in MSs is becoming increasingly versatile. Phase modulation alone can produce lenses, beam deflectors, and holograms, but amplitude modulation allows the creation of spatial filters, structured illumination systems, and complex imaging platforms [65,66,67]. Polarization control enables devices that convert linear to circular polarization or tailor polarization singularities [68]. Dispersion engineering allows MSs to achieve achromatic focusing, multi-wavelength functionality, and group delay control. As MSs continue to evolve, these functional mechanisms will merge, resulting in optical interfaces that act as integrated field processors capable of performing spatial, spectral, and polarization-based transformations simultaneously.

**Figure 1:**
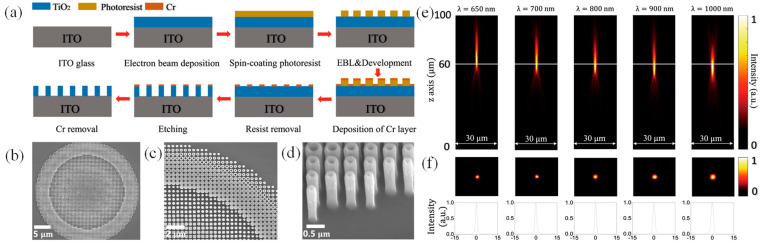
(**a**) Schematic of the fabrication process, showing the highly directional etching used to form the TiO_2_ nanostructures. (**b**,**c**) Top-view SEM images of the achromatic metalens at different magnifications, where four distinct nanopillar geometries are visible. (**d**) Tilt-view SEM image highlighting the structural quality of the fabricated metalens. (**e**,**f**) Measured focal-spot intensity profiles in the x–z and x–y planes at multiple wavelengths; the lower panels show the corresponding radial intensity distributions (solid lines) with fitted curves (dashed lines) [70].

The future of MSs depends critically on fabrication technologies. Electron beam lithography has facilitated the exploration of novel designs, but it cannot support commercialization due to low throughput [69]. Wang et al. demonstrated a broadband, polarization-insensitive TiO_2_ achromatic metalens for near-infrared biological imaging [70]. The device relied on a scalable process that produces high-aspect-ratio nanopillars, ~1.5 µm tall with nearly vertical sidewalls, enabling the required group-delay control for operation across 650–1000 nm. Within this band, the metalens achieved 77–88.5% efficiency and operates with a numerical aperture of 0.24–0.1. The fabrication steps are outlined in Figure 1a. A 1500 nm TiO_2_ layer was deposited by electron-beam evaporation, patterned using PMMA A2 and electron-beam lithography, and transferred into a Cr hard mask via lift-off. Reactive ion etching defined the nanopillars, and the final TiO_2_ structures emerged after Cr removal. Top-view SEM images in Figure 1b,c show 4725 nanopillars with four cross-section types, closely matching the intended design. The tilt-view SEM in Figure 1d confirms ~89°–90° sidewalls and aspect ratios up to ~37.5. Optical characterization is presented in Figure 1e,f. As shown in Figure 1e, all wavelengths from 650 to 1000 nm focus at ~60 µm, matching the designed focal length and NA = 0.24. Figure 1f depicts circular, diffraction-limited focal spots across the band, with Strehl ratios > 0.81 and FWHM deviations < 9%. These results confirm that the fabricated nanopillar array accurately delivers the phase and group-delay profiles required for broadband achromatic performance.

**Figure 2:**
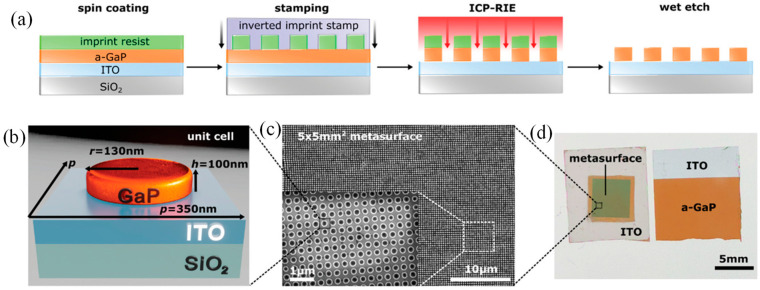
(**a**) Workflow for fabricating the a-GaP MS using nanoimprint lithography, where the patterned polymer layer serves as the etching mask, (**b**) Illustration of the individual MS unit cell, (**c**) SEM micrographs at multiple magnifications showing the large-area nanostructure, (**d**) Photograph comparing two photoelectrodes: one incorporating the imprinted MS (left) and the other consisting of a uniform 100 nm thick a-GaP film, each deposited on a 100 nm thick ITO layer [72].

Nanoimprint lithography presents a path toward scalable manufacturing, while deep ultraviolet lithography may eventually enable MS fabrication in standard semiconductor foundries [71]. Recently, Hutterhofer et al. introduced a scalable imprint-lithography method for fabricating semiconductor photoelectrodes and applied it to amorphous gallium phosphide (a-GaP) [72]. By combining anapole modes with MS lattice resonances, broadband absorption enhancement was achieved. The process offered high throughput and low cost while preserving the engineered photonic response. As shown in Figure 2a, the resist-coated sample was imprinted with the inverted stamp, forming a ~180 nm-thick polymer mask that can be used directly for etching, reducing processing steps. Residual polymer was removed with acetone and isopropanol. Figure 2b–d shows the unit-cell design and SEM images confirming uniform nanostructuring, with only minor radius variations originating from the stamp. The final MS electrode and a planar a-GaP film are shown in Figure 2d. After etching, the exposed ITO around the edges functions as the back contact for both electrodes [72]. Photoelectrochemical measurements under sunlight and hydrogen-evolution conditions showed a 5.7-fold photocurrent increase over a planar film, supported by optical characterization and numerical modeling of both individual nanodisks and the full MS [72].

Additional techniques such as atomic layer deposition and multilayer stacking may allow MSs to expand into three-dimensional architectures, offering enhanced control over optical dispersion and modal composition [73]. Overcoming fabrication challenges will determine whether MSs can transition from specialized components to ubiquitous optical elements in commercial systems. A comparison of plasmonic, MS, and hybrid approaches is presented in Table 1, emphasizing their operational principles and the performance attributes that define their application potential.

## 4. Plasmonic Devices as Drivers of Extreme Performance

Plasmonic devices excel where high field intensity and ultrafast response are essential. Plasmonic modulators demonstrate this potential by achieving significant refractive index changes over extremely small interaction lengths [138]. Transparent conducting oxides in the epsilon near-zero regime support strong index modulation with minimal voltage, enabling modulators that operate at terahertz speeds and potentially even support femtosecond-scale switching [33]. As data rates continue to rise, such modulators could form key building blocks for next-generation on-chip interconnects and optical logic circuits. Coherent optical communication enables the highest data throughput and spectral efficiency, making it well-suited to meet rapidly growing bandwidth demands. It relies on in-phase/quadrature (IQ) electro-optic modulators that encode information in both the amplitude and phase of light. For large-scale integration, these modulators must combine energy efficiency with a compact footprint.

**Figure 3:**
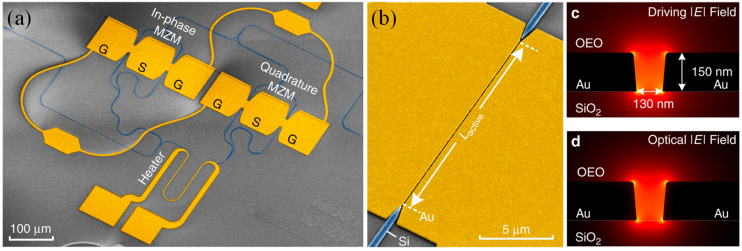
Plasmonic IQ modulators with attojoule-per-bit energy consumption: (**a**) Colorized SEM image of a plasmonic IQ modulator on a silicon platform, incorporating two Mach–Zehnder modulators (MZMs), (**b**) Close-up of the active plasmonic slot waveguide. Light from the silicon waveguide is coupled into the gold slot waveguide via tapered mode converters. The slot is filled with an organic electro-optic material, (**c**,**d**) Cross sections of the plasmonic slot waveguide showing simulated c electrical driving field and d optical field distributions [139].

Heni et al. reported ultracompact silicon-based IQ modulators with an active region of 4 × 25 µm × 3 µm, operable with sub-1 V drivers [139]. The plasmonic–organic hybrid IQ modulators consist of two imbalanced high-speed plasmonic Mach–Zehnder modulators (MZMs) integrated into a silicon photonic Mach–Zehnder interferometer (MZI), as shown in Figure 3a. The phase shift between the I and Q arms was controlled either by a thermo-optic phase shifter or by wavelength tuning enabled by the MZI imbalance. DC biases independently set the operating points of the two MZMs. Each MZM contained a 15 or 20 µm plasmonic slot waveguide with a 130 nm slot width (Figure 3b). The metal–insulator–metal (MIM) slot was filled with the organic electro-optic (OEO) composite HD-BB-OH/YLD124. In the active region, the electrical RF field and the optical mode were both tightly confined to the slot and exhibited nearly complete spatial overlap (Figure 3c,d). They achieved exceptionally low electrical energy consumption: 0.07 fJ bit^−1^ at 50 Gbit s^−1^, 0.3 fJ bit^−1^ at 200 Gbit s^−1^, and 2 fJ bit^−1^ at 400 Gbit s^−1^. These performance metrics highlight the potential of such devices for both long-haul and short-reach coherent communication systems.

**Figure 4:**
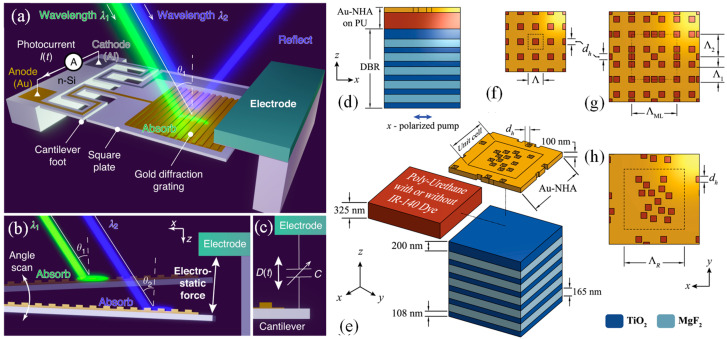
Principle of the electromechanically reconfigurable plasmonic photodetector, (**a**) Schematic of the device featuring a plasmonic structure with a gold diffraction grating, (**b**) Electromechanical angular scanning enabling wavelength-dependent photodetection, (**c**) Capacitive coupling between the fixed electrode and the cantilever, generating a periodic displacement current [143], (**d**) Schematic of the Tamm plasmon (TP) device comprising a Au nanohole array (Au-NHA) on a polyurethane (PU) layer placed atop a DBR, illuminated from below along the + z direction, (**e**) Exploded view with key structural parameters. The PU layer can be either undoped—serving as a passive resonant cavity—or doped with IR-140 dye to form a Tamm plasmonic laser (TPL), (**f**–**h**) Top-view illustrations of three NHA configurations and their corresponding unit-cell lengths: (**f**) a regular NHA with period Λ, (**g**) a multilattice (ML) NHA with additional periods Λ_1_ and Λ_2_, and (**h**) a unit cell containing randomly distributed holes. All Au-NHA holes are square with a side length [147].

Photodetectors that incorporate plasmonic structures utilize localized resonances to concentrate optical energy into the semiconductor region or generate hot electrons that contribute directly to photocurrent [140,141]. These mechanisms can extend detection bandwidth, enhance responsivity, and reduce detector volume. Plasmonic detectors could be integrated into multispectral imaging systems, biosensing platforms, and hyperspectral cameras, especially when combined with MSs that provide spectral filtering or beam shaping [4,142]. Oshita et al. presented a plasmonic photodetector that reconfigures its spectral responsivity through electromechanical deformation rather than electrical bias control [143]. The photodetector consisted of an n-type silicon cantilever with a plasmonic gold diffraction grating on its surface and a nearby electrode (Figure 4a). Applying a voltage between the electrode and the cantilever generates an electrostatic force that drives the cantilever into resonance (Figure 4b), thereby changing the incident angle θ and reconfiguring the plasmonic resonance. Dual zigzag support structures reduce stiffness and increase the angular scanning amplitude.

When light of an appropriate wavelength strikes the grating at the resonant angle, surface plasmon resonance (SPR) enhances absorption. The absorbed energy excites electrons in the gold, enabling them to overcome the Schottky barrier at the Au/n-Si interface. These electrons are collected as a photocurrent I(t) between the gold anode and aluminum cathode. Because the incident angle is periodically modulated, the detector’s spectral response is scanned in time. However, capacitive coupling between the cantilever and the electrode (Figure 4c) introduces a displacement current D(t), which appears as a periodic background signal due to repeated charging and discharging of the capacitor. This approach produced clear peak shifts across 1250–1310 nm. To highlight potential applications, near-infrared spectroscopy was also demonstrated using the device. Because the structure enables sub-bandgap detection in silicon via a Schottky junction, this photodetector is a promising candidate for compact near-infrared spectrometers integrated into industrial silicon imaging systems [143].

Plasmonic nanolasers offer a route to coherent light generation at scales far smaller than traditional optical cavities [144,145]. By confining electromagnetic fields within volumes comparable to the cubic wavelength in matter or even smaller, nanolasers can interact strongly with emitters and achieve threshold reductions [146]. Their small footprint makes them compatible with dense photonic integration and suggests they may become key components of optical interconnect networks or photonic neural architectures [29]. Extraordinary optical transmission (EOT) in metal nanohole arrays (NHAs) and Tamm plasmon (TP) states have been studied since their discoveries in 1998 and 2007, respectively. Both phenomena underpin a wide range of plasmonic devices, including absorbers, lasers, and narrowband filters. Although the influence of hole size and geometry on EOT has been well documented, the corresponding effects on TP structures incorporating metal NHAs remain less explored—particularly the role of controlled randomness in the hole arrangement.

Shahid et al. systematically varied both the hole size and spatial distribution in metal NHAs and examined the resulting changes in EOT and Tamm resonances [147]. Three configurations were investigated: a bare NHA, a passive TP cavity, and a TP laser. The device structures and excitation direction are shown in Figure 4d–h. Figure 4d,e illustrate the Tamm plasmon (TP) cavity, which consists of a 100 nm-thick perforated Au film on top of a PU layer. The hole-array configurations in Figure 4f–h are applied to the Au layer of the TP cavity shown in Figure 4e. When the PU layer was doped with IR-140 dye molecules, the passive TP structure became an active Tamm plasmon laser (TPL). The characteristics of the Au nanohole array (NHA) were controlled by adjusting the hole arrangement, the unit-cell length (Λ), and the hole size (d_h_). Figure 4f–h present the three NHA configurations investigated. The first is a simple periodic square array with period Λ. The second, shown in Figure 4g, is a multilattice (ML) arrangement composed of two concentric square arrays with periods Λ_1_ and Λ_2_. The third configuration, shown in Figure 4h, consists of nanoholes placed randomly within each repeating unit cell. As periodicity increases, multiple Tamm resonances emerge; however, these resonances disappear once the array transitions to a pseudo-periodic random configuration, defined as holes placed randomly within a repeating square unit cell. The appearance of multiple resonances was attributed to dispersion-line folding in periodically patterned TP cavities. Dispersion calculations further clarify the origin and behavior of these resonance features in both transmission and lasing emission spectra [147].

Plasmonic waveguides, although limited by propagation loss, remain valuable for routing optical energy within extremely compact regions [77,92]. Hybrid waveguides that combine dielectric and plasmonic confinement can balance loss and confinement to achieve functional pathways for dense circuit architectures [81,148]. As the need for optical interconnect density increases, plasmonic waveguides may provide the only viable solution for submicron-scale wiring.

## 5. MS Photonic Devices as System-Level Disruptors

MS devices have advanced beyond individual optical components and are now poised to reshape entire systems [115,126,135]. Beam steering MSs, for example, can replace bulky mechanical systems in LiDAR by imposing electronically controlled phase gradients. This approach allows rapid scanning speeds, reduced power consumption, and compact form factors suitable for consumer electronics and autonomous navigation systems [149]. Metalenses illustrate the transformative potential of MSs by collapsing the complexity of traditional lens systems into thin, planar layers [11]. These lenses can be engineered for specific spectral bands, for broad achromatic operation, or for insensitivity to polarization. As computational imaging techniques mature, metalenses may be integrated with algorithms to produce imaging systems that achieve superior performance with minimal hardware [115].

**Figure 5:**
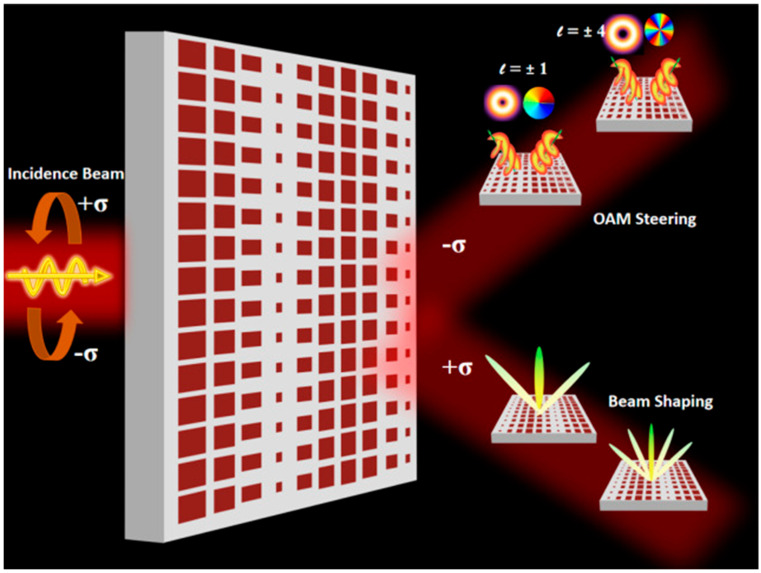
Schematic illustration of the various spin-switchable functionalities enabled in the proposed design by altering the handedness of the incident light [150].

Recently, Ullah et al. demonstrated an all-dielectric coding MS that advances the understanding of spin-dependent light–matter interactions and enables spin-switchable wavefront manipulation [150]. Their design, composed of Si nanobricks arranged in a square lattice, exploits tailored coding patterns to simultaneously modulate the geometric and propagation phases at 780 nm. This capability allows efficient spin-dependent wavefront control, enabling functionalities such as generating optical vortex beams with various orbital angular momentum (OAM) states and performing beam-shaping operations that split and steer the incident beam at prescribed angles. Figure 5 presents the schematic configuration of the proposed 2-bit/1-bit spin-selective coding MS. When circularly polarized (CP) light with handedness ±σ is incident normally, the MS directs the output into the cross-polarized channel, generating either an OAM-carrying structured beam or a split beam depending on the input spin state. Under right-handed circular polarization (RCP, +σ), the MS functions as a vortex-beam generator, producing distinct OAM modes. In contrast, left-handed circular polarization (LCP, −σ) induces controlled beam splitting at a designed angle. These functionalities arise from the precise engineering of the coding patterns and the hybrid 2-bit/1-bit encoding, which enables flexible and independent manipulation of the transmitted wavefronts. Convolution-based operations further enhance the versatility of the MS, supporting more complex and multichannel optical manipulations. Collectively, this strategy provides a simple yet powerful framework for developing high-capacity encrypted communications, quantum information systems, and advanced non-invasive imaging technologies [150].

**Figure 6:**
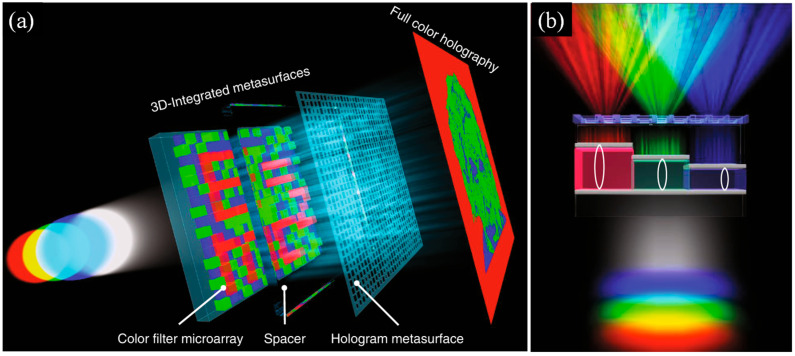
3D-Integrated MSs for Full-Color Holography: (**a**) The exploded diagram shows the device architecture. A color-filter microarray forms a visible microprint under white-light illumination, while the underlying MS encodes holographic phase information. When illuminated with red, green, and blue lasers, the structure produces three independent far-field holograms that can be combined to form a full-color image, (**b**) The front view shows three micro-units composed of metal–dielectric–metal Fabry–Pérot cavity filters. Each filter transmits only the laser wavelength closest to its resonance, directing the selected light to the hologram MS and generating three separate monochromatic greyscale holograms [154].

Holographic MSs extend this concept by offering complete control over the structure of optical wavefronts [151,152]. They can generate three-dimensional holograms, encode spatial information into beams, or perform optical transformations that mimic the functions of neural networks. Such holographic processors could one day serve as analog accelerators for machine learning workloads [153]. Hu et al. presented a 3D-integrated MS device that stacked a hologram MS with a monolithic Fabry–Pérot cavity color-filter microarray, enabling simultaneous low-crosstalk, polarization-independent, high-efficiency full-color holography and color microprinting [154]. This dual-function platform offers a new route for applications such as data storage, security encoding, color display technologies, and information processing. The 3D integration strategy can be further expanded by incorporating additional functional MS layers, such as polarizers or metalenses to create multifunctional flat optical systems. Figure 6a shows the 3D-integrated MS, consisting of a stepwise MDMFP color-filter array and a hologram MS. The metal–dielectric–metal Fabry–Pérot filters, formed by varying the dielectric thickness, provide high transmission, a wide color gamut, and narrow linewidths, superior to plasmonic color filters [154].

The hologram MS, made of isotropic dielectric nanostructures, imparts the required phase profile for high-quality far-field holography. As illustrated in Figure 6b, under RGB laser illumination, each wavelength passes only through the filter whose resonance closely matches it, then reaches the hologram layer to form three independent monochromatic greyscale holograms. Combining these channels yields a full-color hologram. The device simultaneously encodes holographic information and color microprint patterns within the arranged filter array. This 3D integration offers several advantages over wavelength-multiplexed plasmonic and PB-phase MS holography: (i) higher transmission and diffraction efficiency due to FP cavity filters and dielectric nanostructures; (ii) reduced inter-channel crosstalk from the narrow FP resonances; (iii) polarization-independent operation; and (iv) straightforward fabrication using standard EBL and metal deposition without pattern-transfer steps [154].

**Figure 7:**
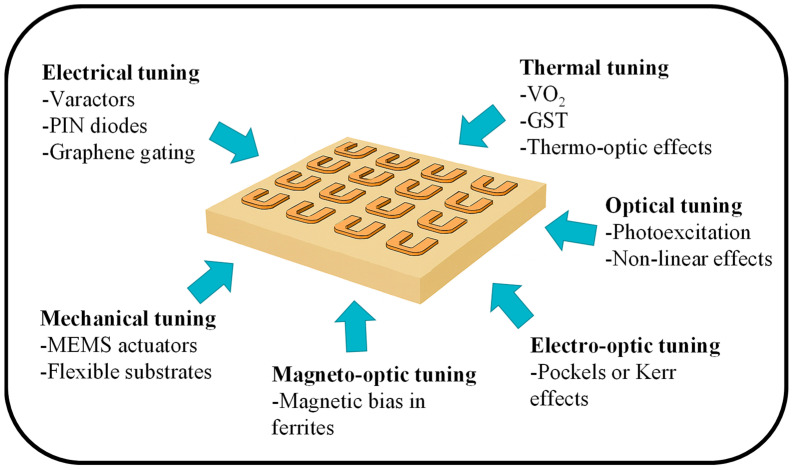
Tuning mechanisms for reconfigurable MSs.

Reconfigurable MSs represent one of the most exciting directions for future research [11,116,117]. By incorporating materials such as phase change alloys, liquid crystals, graphene, or mechanical actuation layers, MSs can dynamically alter their optical response [155,156,157]. These platforms can tailor electromagnetic wave propagation through a wide range of tuning mechanisms that modify their effective optical, electrical, or geometric properties (Figure 7). Electrical tuning uses components including varactors, PIN diodes, and graphene gating to adjust the response by altering circuit parameters or carrier density [158]. Thermal tuning, enabled by phase-change materials such as VO_2_ or GST and by thermo-optic effects, modulates the refractive index through controlled heating [159]. Optical tuning relies on photoexcitation or nonlinear optical processes to modify carrier concentration or refractive index using incident light [160,161]. Mechanical tuning, implemented with MEMS actuators or flexible substrates, directly alters the physical geometry of the meta-atoms [162]. Additional mechanisms such as electro-optic tuning, which exploits the Pockels or Kerr effects, and magneto-optic tuning, which uses magnetic bias in ferrites, provide high-speed or nonreciprocal control [163]. Collectively, these mechanisms offer powerful and versatile pathways for achieving dynamic beam steering, tunable absorption, reconfigurable holography, and adaptive wavefront engineering.

## 6. Hybrid Plasmonic and Photonic Architectures for Planar Optical Systems

Hybrid photonic plasmonic systems provide an effective pathway for combining the complementary strengths of dielectric photonics, plasmonics, and MSs within planar optical platforms. Dielectric components offer low-loss signal distribution and stable optical functionality, while plasmonic elements enable extreme field confinement, ultrafast response, and enhanced light-matter interaction within deeply subwavelength regions [55,80,164,165]. By incorporating plasmonic functionality only where strong interaction is required, hybrid systems achieve high performance without the efficiency penalties associated with fully plasmonic architectures.

**Figure 8:**
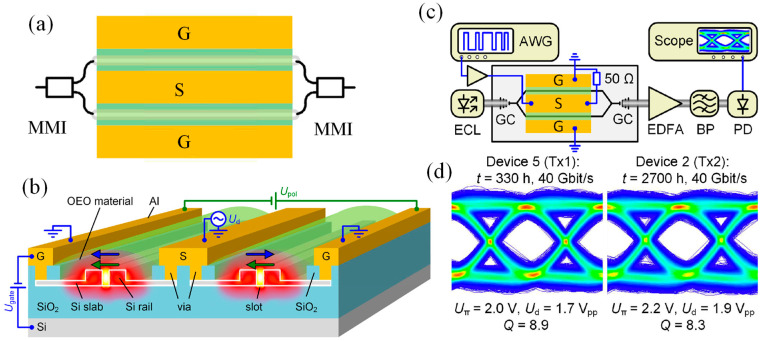
SOH modulator concept. (**a**) Top view of the SOH MZM with GSG transmission line and MMI couplers, (**b**) Perspective view showing the Si slot waveguides, EO-filled slot, poling process, and push–pull operation, (**c**) Setup for generating and detecting 40-Gbit/s signals using the SOH modulator, (**d**) 40-Gbit/s eye diagrams for devices after 330 h and 2700 h of high-temperature storage, showing ~10% drive-voltage increase for the aged device [168].

Hybrid modulators serve as a representative example of this approach. In such devices, localized plasmonic regions are incorporated within otherwise dielectric guiding structures, enabling large modulation efficiency over short interaction lengths [166,167]. Kieninger et al. demonstrated long-term thermally stable silicon organic hybrid modulators that satisfy Telcordia high temperature storage requirements [168]. These results highlight the suitability of hybrid architectures for practical and reliable optical systems. The silicon organic hybrid Mach-Zehnder modulator shown in Figure 8 illustrates the operating principles of this approach. Figure 8a presents the top view of the device, showing the Mach Zehnder interferometer geometry with multimode interference couplers for optical splitting and recombination, along with the ground signal ground transmission line used for electrical driving. Figure 8b depicts the cross section of each interferometer arm, where a silicon slot waveguide filled with an electro-optic polymer concentrates the radio frequency electric field within a nanoscale region. This configuration enables strong overlaps between the electrical and optical fields, resulting in efficient phase modulation while maintaining low loss dielectric propagation. Figure 8c shows the high-speed transmission configuration used to evaluate device performance, illustrating how the modulated optical signal is generated, amplified, and detected. Figure 8d presents representative eye diagrams at forty gigabits per second after extended thermal storage, demonstrating stable high speed operation and confirming the robustness of the hybrid design [168].

**Figure 9:**
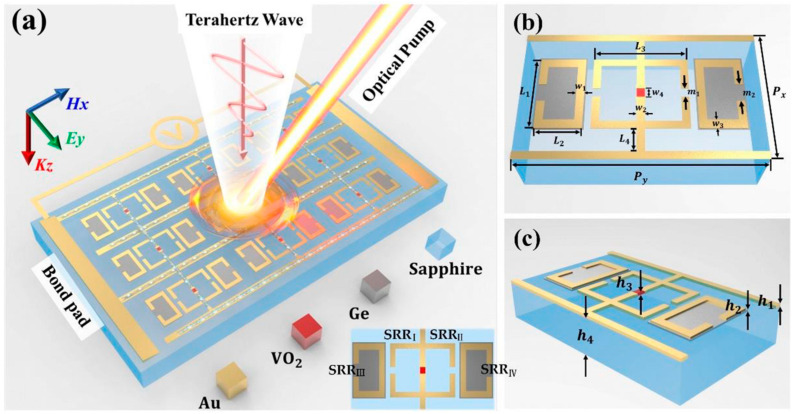
Schematic design and operating mechanism of the proposed hybrid MS. (**a**) Conceptual illustration of the MS, where the phase-change material VO_2_ is electrically driven, and the semiconductor Ge is modulated by optical pumping. (**b**,**c**) Geometric configuration and detailed dimensional parameters of the unit cell [169].

Hybridization also extends naturally to MS-based platforms, where plasmonic and active material responses can be combined at the meta-atom level. Figure 9 illustrates a terahertz hybrid MS that integrates multiple control mechanisms within each meta-atom [169]. Figure 9a shows the unit cell architecture, in which phase change material and semiconductor components are co-located to enable electrically and optically driven modulation. Figure 9b illustrates the simulated or measured electromagnetic response of the MS under different external stimuli, demonstrating independent tuning channels. Figure 9c presents system-level functionality in the form of switching or logic operations enabled by multi-field control. This example highlights how hybrid MSs move beyond passive wavefront shaping to support programmable and multifunctional optical behavior [169].

Efficient coupling between dielectric and plasmonic regions remains a central challenge due to mismatched modal profiles and dispersion characteristics [55,80]. Design strategies such as tapered transitions, slot-based confinement, and impedance-matching structures enable controlled energy transfer over short distances, allowing plasmonic regions to function as localized processing nodes embedded within larger planar optical systems.

Despite their advantages, hybrid photonic plasmonic systems face challenges related to optical loss, thermal management, and fabrication tolerance [165]. Heat generated by plasmonic elements can influence nearby dielectric or MS structures, while nanoscale fabrication variations can affect coupling efficiency and device reproducibility. Continued advances in alternative plasmonic materials, thermal engineering, and scalable nanofabrication techniques are therefore essential.

From a system perspective, hybrid platforms enable simultaneous optimization of footprint, efficiency, speed, and functionality. Compared to purely dielectric devices, hybrid systems offer enhanced modulation efficiency and access to ultrafast and nonlinear optical responses [101,170]. Compared to fully plasmonic implementations, they provide improved stability and reduced loss. As a result, hybrid photonic plasmonic architectures represent a practical pathway toward compact, multifunctional, and programmable planar optical systems that bridge nanoscale physics and real-world applications [5,64,171,172,173].

## 7. Applications and Emerging Technologies That Will Drive Adoption

The convergence of plasmonics and MSs aligns closely with several emerging technological trends [174]. Optical computing stands at the forefront, requiring high-speed operations and parallel processing capabilities [175,176]. MS-based optical neural networks can process information at the speed of light by performing matrix multiplications in free space, while plasmonic nonlinearities may offer the necessary activation functions for all optical deep learning systems [175].

**Figure 10:**
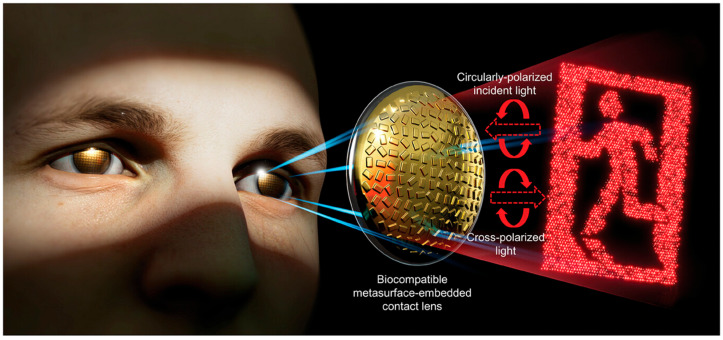
Schematic illustration of an MS-integrated contact lens for holographic light projection. Upon illumination with circularly polarized light, the all-metallic MS reflects and reconstructs the designed holographic image [178].

Augmented reality (AR) and virtual reality (VR) demand compact, lightweight, and high-performance optical systems [5]. MSs can produce thin lens stacks, holographic combiners, and tunable beam shaping devices that are essential for next generation wearable displays. Integrating plasmonic sensors into these systems could provide simultaneous health monitoring or environmental sensing [177]. Contact lenses play a central role in vision correction and are increasingly regarded as a promising platform for AR displays through the incorporation of electronic and optical functionalities. MSs provide rich optical control within an ultrathin footprint, making them attractive for compact imaging and display applications. Despite this potential, integrating MSs into contact lenses remains difficult due to the limited biocompatibility of conventional imprinting materials and structural deformation arising from swelling and contraction on hydrated surfaces. Ko et al. presented a biocompatible transfer strategy that employs hyaluronic acid (HA) as a soft mold to realize MS integration on contact lenses and enable holographic light projection [178]. A high-efficiency metahologram was achieved using all-metallic three-dimensional meta-atoms with rectangular anisotropy and a reflective metallic backplane. Figure 10 illustrates the schematic of an MS-integrated contact lens. In the reflection mode, the MS-engineered wavefront propagates into the far field, where it reconstructs the target holographic image. The phase-only computer-generated hologram (CGH) was obtained using the Gerchberg–Saxton (GS) algorithm based on iterative Fourier transforms. To efficiently realize the retrieved phase profile, the MS consists of uniformly sized meta-atoms with spatially varying in-plane rotation angles, enabling phase control through the Pancharatnam–Berry (PB) mechanism. By leveraging the PB phase, the reflected wavefront acquires a spatially tailored phase distribution while maintaining a nearly uniform amplitude. To suppress wrinkling and maintain structural integrity during transfer onto soft, wettable lenses, a corrugated metallic layer on the HA mold was reinforced with a SiO_2_ capping layer. This biocompatible transfer approach opens a pathway for embedding diverse optical elements, including holograms, lenses, and gratings onto contact lenses, advancing AR displays and human–computer interaction technologies [178].

Sensing and spectroscopy represent another major application space [171,172,173]. Plasmonic hotspots greatly enhance Raman scattering, enabling the detection of molecular signatures at extremely low concentrations. MS-based absorbers and filters allow for compact spectrometers that operate across visible, infrared, and terahertz bands. These platforms can support portable diagnostic devices, environmental sensors, and chemical analysis systems [179]. Bound states in the continuum (BICs) are nonradiative optical modes with strong field confinement and growing relevance in nanophotonics. They are commonly classified as symmetry-protected or accidental BICs, depending on the radiation suppression mechanism. While symmetry-protected BICs are typically controlled by a single structural parameter, accidental BICs rely on complex mode coupling, making their design and application more challenging. Li et al. developed an accidental BIC MS based on periodic H-shaped Si_3_N_4_ nanopillars and evaluated its refractive index sensing performance through both wavelength shift and intensity modulation [180]. The sensor demonstrated high sensitivity, achieving 501 nm/RIU in wavelength-based detection and 9.35 × 10^4^ counts/RIU in intensity-based detection, and shows strong potential for biodetection applications. These results provide new insights into mode coupling in BIC systems and introduce an effective dual-channel approach for high-sensitivity optical sensing [180].

**Figure 11:**
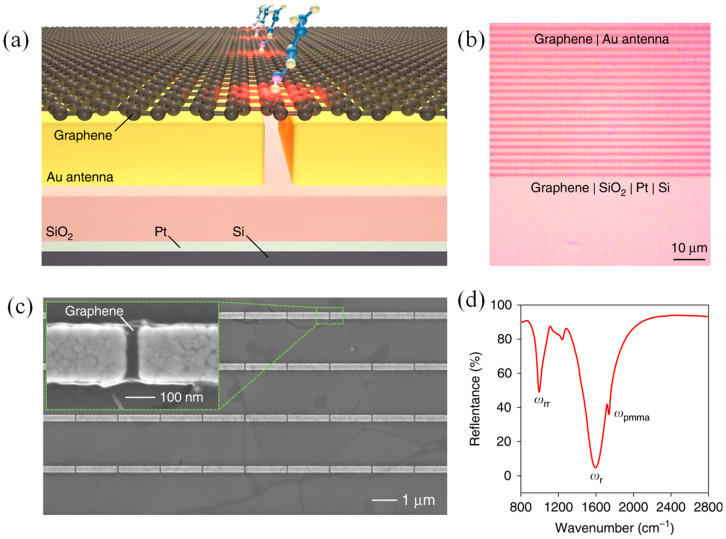
(**a**) Schematic illustration of the graphene–metal MS, where small analyte molecules are adsorbed onto suspended monolayer graphene. (**b**) Optical microscopy image of a fabricated device, confirming the uniform and continuous coverage of graphene across a large area. (**c**) SEM image of graphene-coated nanorod antennas; the inset highlights a single antenna gap bridged by suspended graphene. (**d**) Measured reflectance spectrum showing the dominant plasmonic resonance (ω_r_) around 1500 cm^−1^, a secondary resonance dip (ω_rr_) near 1000 cm^−1^, and the PMMA absorption feature (ω_PMMA) at approximately 1700 cm^−1^ [173].

Optical biosensors enable quantitative analysis and molecular structural identification, yet conventional refractive-index-based methods often lack the sensitivity needed to detect low-molecular-weight analytes. Although non-optical platforms such as field-effect transistors offer higher sensitivity, they do not provide molecular fingerprinting capabilities. To address these limitations, Zhu et al. developed mid-infrared biosensors based on optical conductivity, enabling sensitive and quantitative detection of small molecules while enhancing their spectroscopic signatures [173]. These sensors employed a hybrid MS composed of monolayer graphene integrated with metallic nanoantennas, combining plasmonic, electronic, and spectroscopic functionalities. Molecular adsorption induces carrier doping in graphene, which can be optically detected with high sensitivity. Because the resulting resonance shifts are directly linked to graphene carrier density, the platform enables reliable quantification of molecular interactions and is insensitive to carrier mobility degradation. Figure 11 shows the structure and optical response of the hybrid MS.

As illustrated in Figure 11a, the device consisted of a periodic array of Au nanorod antennas coated with monolayer graphene and separated from a Pt back reflector by a SiO_2_ spacer, forming an optical cavity. The optical micrograph in Figure 11b confirms uniform graphene coverage over a large area, while the SEM image in Figure 11c reveals graphene conformally covering the nanorods and remaining suspended across 30 nm gaps, as highlighted in the inset. The reflectance spectrum in Figure 11d exhibits two mid-infrared resonance features arising from plasmon–phonon coupling: a primary plasmonic resonance near 1500 cm^−1^ (ω_r_) and a secondary dip around 1000 cm^−1^ (ω_rr_), along with a PMMA-related absorption peak near 1700 cm^−1^. In addition, the MS supported surface-enhanced infrared spectroscopy, allowing detection of sub-nanometer molecular monolayers and affinity-based glucose sensing down to 200 pM, as well as enhanced infrared fingerprinting of trace biomolecules and polymers [173].

Communications technology continues to push for higher bandwidth and lower latency [181]. MS-based wavelength management and plasmonic high-speed modulators can significantly enhance the performance of optical interconnects [182,183]. Liu et al. presented a hybrid optical modulator formed by integrating a graphene sheet with a dielectric MS that supports a pronounced toroidal resonance [184]. The MS was composed of a pair of mirror-imaged, asymmetric silicon split-ring resonators capable of sustaining a strong toroidal dipole mode characterized by an ultra-narrow spectral linewidth (~0.77 nm), a high-quality factor (~1702), and nearly complete resonance contrast. Numerical simulations revealed that incorporating graphene enables effective control of the resonance transmission by tuning its Fermi level. A maximum modulation depth of approximately 78% was obtained, demonstrating the strong modulation capability of the proposed structure. In addition, the influence of resonator asymmetry on the toroidal response was systematically analyzed, along with the dependence of modulation efficiency on graphene parameters. These findings highlight the potential of the graphene–dielectric MS platform for applications in optical modulation, filtering, and biochemical sensing [184].

**Figure 12:**
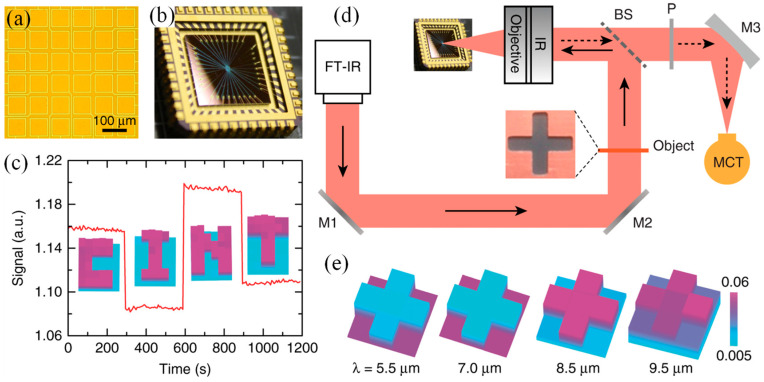
Hybrid graphene–MS spatial light modulator and single-pixel imaging demonstration. (**a**) Optical micrograph of the active region, showing a 6 × 6 array of independently addressable functional pixels, (**b**) Photograph of the fabricated device after wire bonding to a chip carrier, (**c**) Measured spatial reflection patterns forming the word “CINT” at a wavelength of 8.3 μm, obtained by selectively applying gate voltages of −3 V (OFF) and +7 V (ON) to individual pixels. The insets show the corresponding mask patterns acquired by raster scanning, where only one pixel is switched ON at a time while all others remain OFF (purple denotes the ON state). Variations in the applied SLM patterns lead to distinct changes in the detected single-pixel signal intensity, (**d**) Schematic of the single-pixel imaging setup incorporating the hybrid graphene–MS SLM, including flat mirrors (M1, M2), a beam splitter (BS), a polarizer (P), a parabolic mirror (M3), and a mercury–cadmium–telluride (MCT) single-pixel detector, (**e**) Reconstructed images of a cross-shaped target obtained using a raster-scan measurement matrix at wavelengths of 5.5 μm, 7 μm, 8.5 μm, and 9.5 μm, demonstrating broadband imaging capability [185].

Furthermore, Zeng et al. exploited the synergistic integration of graphene with engineered MSs to realize a free-space mid-infrared modulator that operates at gigahertz frequencies, requires low driving voltages, and functions at room temperature [185]. By further patterning the hybrid graphene–MS structure into addressable pixels, a prototype spatial light modulator capable of high-frame-rate single-pixel imaging was demonstrated, offering performance improvements by several orders of magnitude compared with traditional liquid-crystal or micromirror-based devices. These results paved the way for advanced infrared wavefront manipulation, where rapid temporal and spatial modulation is essential for next-generation photonic technologies [185]. Figure 12 illustrates the operation and imaging performance of the hybrid graphene–MS spatial light modulator (SLM). The fabricated prototype consists of a 6 × 6 array of electrically isolated functional pixels, as shown in Figure 12a,b. Each pixel can be independently switched between an “ON” and “OFF” state by applying gate voltages of +7 V and −3 V, respectively, enabling programmable spatial modulation of mid-infrared light.

The device functionality was first characterized through raster scanning of individual pixels. As shown in the insets of Figure 12c, predefined mask patterns forming the letters “CINT” were clearly resolved by sequentially activating one pixel at a time while keeping all others in the OFF state. These measurements, performed at a wavelength of 8.3 μm, demonstrated accurate spatial encoding by the MS SLM. When the pixels corresponding to each mask are switched ON simultaneously, the reflected signal exhibits distinct real-time variations as different masks are applied, as indicated by the red trace in Figure 12c. The single-pixel imaging capability of the SLM was demonstrated using the experimental configuration shown schematically in Figure 12d. Reconstructed images of a cross-shaped object at different wavelengths are presented in Figure 12e, confirming broadband imaging functionality. Higher image contrast was observed at wavelengths of 7 μm and 8.5 μm, whereas reduced contrast appeared at 5.5 μm and 9.5 μm, reflecting the wavelength-dependent modulation efficiency of the device. Together, Figure 12a–e validate the feasibility of the hybrid graphene–MS SLM for high-speed, programmable mid-infrared imaging applications [185].

## 8. Core Challenges Driving the Research Strategy

**Figure 13:**
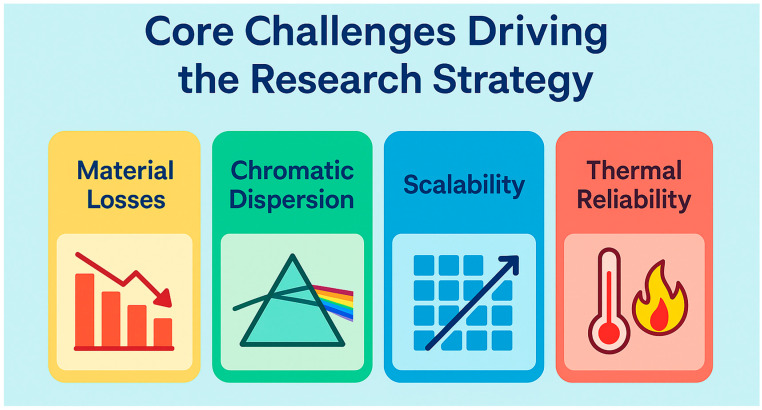
Key Technical Challenges Limiting Scalable MS and Plasmonic Technologies.

Although the future appears promising, several grand challenges stand between current technologies and large-scale deployment. As summarized in Figure 13, advancing MS and plasmonic platforms toward scalable, high-performance operation requires addressing four primary hurdles: (i) intrinsic material losses that limit efficiency in plasmonic systems, (ii) chromatic dispersion that constrains broadband and achromatic functionality, (iii) fabrication scalability necessary for wafer-level patterning with nanoscale precision, and (iv) thermal reliability issues arising from optical field–induced heating and repeated phase-change cycling.

From a manufacturing perspective, scalable preparation technologies capable of balancing nanoscale accuracy with high throughput are rapidly emerging [186]. Deep ultraviolet lithography, particularly 193 nm immersion processes, offers excellent critical dimension control, overlay accuracy, and compatibility with CMOS foundries, making it well-suited for wafer-scale MS and plasmonic device fabrication [187,188]. Nanoimprint lithography provides a complementary approach, enabling cost-effective, high-throughput replication of dense nanostructures over large areas and has already been adopted for commercial metalens production [189,190]. Hybrid fabrication strategies that combine optical lithography for alignment-sensitive layers with nanoimprint techniques for large-area patterning further enhance manufacturability, suggesting a realistic pathway toward mass production of plasmonic and MS-based optical systems [35,191].

Loss in plasmonic materials remains a fundamental issue, particularly for applications requiring high efficiency [32,192]. Advances in material synthesis, epitaxy, and hybrid mode engineering may mitigate these losses, but completely overcoming them remains difficult [193]. MSs face their own limitations in chromatic dispersion, which restricts broadband functionality and achromatic imaging performance [194]. Scalability of fabrication is another major barrier. To integrate MSs into consumer electronics or large area optical systems, manufacturing processes must transition toward wafer-scale patterning without sacrificing nanoscale precision [195,196]. Furthermore, thermal reliability presents concerns for both plasmonic and phase change-based MSs, as repeated cycling or sustained field intensity can degrade material performance [197,198]. These challenges reflect not only technological hurdles but also opportunities for interdisciplinary innovation. The combined expertise of materials scientists, nanofabrication engineers, optical physicists, and computational designers will be essential for shaping the next generation of integrated optical systems.

Beyond the challenges discussed above, several open issues remain insufficiently explored at a fundamental and system level. In particular, the long-term stability, aging behavior, and failure mechanisms of densely integrated plasmonic and MS structures under realistic electrical, thermal, and optical operating conditions are not yet well understood. Equally important is the absence of unified system-level design methodologies that explicitly account for cross-domain interactions among optics, electronics, thermal transport, and control circuitry. Finally, there is a growing need for predictive multiscale modeling frameworks that can bridge nanoscale electromagnetic response with macroscopic system performance, reliability, and manufacturability. Addressing these issues will be critical for the transition from proof-of-concept demonstrations to robust, scalable photonic technologies.

An additional challenge that becomes increasingly critical at the system level is balancing peak device performance with power consumption and thermal management [199]. While plasmonic and MS devices often demonstrate record speed, compactness, or field confinement, practical deployment requires optimization of energy-per-operation rather than isolated performance metrics [200]. In many cases, reduced device length, lower capacitance, and strong light–matter interaction enable overall energy efficiency despite intrinsic optical loss. Hybrid architectures that localize high-speed or high-field functionality to specific regions, while relying on low-loss dielectric routing elsewhere, provide a practical pathway for managing thermal load [201,202]. As a result, performance, power dissipation, and thermal stability must be treated as coupled design variables in future integrated photonic systems rather than optimized independently.

## 9. Future Vision: Roadmap for Next-Generation Plasmonic and MS Photonics

The future of planar optical systems is increasingly shaped by concrete experimental demonstrations that translate plasmonic and MS concepts from theoretical constructs into functional devices and system-level components [123]. A growing body of work reveals a clear trajectory toward optical platforms that are flat by design, programmable in functionality, and tightly coupled to electronic drivers and computational frameworks, signaling a fundamental shift in how optical hardware is conceived and implemented [203,204]. Rather than relying on bulk components or extended propagation paths, future optical systems emphasize interface-engineered functionality, where light manipulation occurs within subwavelength regions and planar layers.

Metalens-based imaging platforms provide one of the most visible indicators of this transition. Commercial demonstrations have shown that a single MS lens can replace complex stacks of refractive elements in compact sensing modules, while remaining compatible with semiconductor manufacturing and delivering performance suitable for depth sensing and biometric applications [205]. Academic and industrial efforts have further extended this concept to compact infrared imaging systems for extended reality headsets and multi-wavelength metalenses for aerial and mobile cameras [122]. These developments outline a realistic pathway toward optical stacks for cameras, microscopes, and head-mounted displays that achieve high performance within thicknesses of only hundreds of nanometers to a few micrometers.

A similar evolution is occurring in beam steering and ranging technologies. MS-based beam steering using liquid crystal loading and high-index nanoantenna arrays has demonstrated solid-state operation without mechanical motion and with angular coverage sufficient for automotive lidar [149]. Emerging commercial platforms further validate this approach by introducing programmable MS chips capable of large-angle beam steering controlled entirely through software, already integrated into three-dimensional ranging modules [206]. These results suggest that future depth sensing, free space communication, and adaptive illumination systems will increasingly rely on flat programmable apertures rather than mechanically scanned optics or phased arrays.

Intelligent photonic surfaces represent another major pillar of the roadmap. Diffractive and MS-based optical neural networks have demonstrated that passive planar layers can perform classification and inference tasks directly in the optical domain [207]. Subsequent implementations using MSs with tailored phase profiles have enabled image recognition, digit classification, and multiplexed optical processing in both near-field and far-field configurations, including architectures that exploit geometric phase control and polarization multiplexing [208]. More recent proposals extend this concept toward all optical convolutional neural networks in which MSs implement convolutional operations and diffractive layers act as optical decoders, pointing toward physically implemented machine learning systems that operate at the speed of light [209]. These advances illustrate how MSs can evolve from static optical components into adaptive information processing layers co-designed with electronic learning and control algorithms.

On the plasmonic side, hybrid photonic plasmonic devices provide a complementary pathway toward ultrafast and energy-efficient active functionality at deeply subwavelength scales. Hybrid modulators incorporating transparent conducting oxides operating in the epsilon near zero regime have demonstrated micrometer-scale footprints, femtojoule per bit energy consumption, and multi-gigahertz modulation bandwidths [210]. Related architectures that integrate indium tin oxide or similar materials within slot waveguides or asymmetric coupling structures further enhance light-matter interaction while maintaining manageable insertion loss [93]. Proposals combining transparent conducting oxides with graphene indicate that two-dimensional materials will play an increasingly important role in future active devices by enabling stronger tunability and reduced driving voltages [211]. Collectively, these results support a vision in which plasmonic elements function as localized switching and modulation nodes embedded within broader planar optical systems rather than as standalone guiding platforms.

Reconfigurable MSs form an additional cornerstone of the roadmap. Platforms based on phase change materials, mechanical actuation, and electro-optical tuning enable dynamic control over phase, amplitude, and beam direction [212]. Low-loss phase change materials such as antimony selenide have enabled rewritable on-chip MSs with strong refractive index contrast and nonvolatile operation in technologically relevant spectral bands [213,214]. Mechanically tunable and liquid crystal-based MSs have demonstrated real-time beam steering and adaptive wavefront control, directly addressing the needs of lidar, adaptive optics, and dynamic imaging systems [215,216]. As these technologies mature, intelligent photonic surfaces capable of real-time optimization through sensor feedback and machine learning based controllers become increasingly feasible.

Taken together, these developments outline a coherent roadmap for next-generation planar photonics. Continued progress in low-loss plasmonic materials and optimized hybrid geometries is required to push modulators and switches into regimes where they outperform purely dielectric devices in both energy efficiency and footprint [136,217]. MSs must advance toward broadband and multifunctional operation, as demonstrated by multicolor metalenses and folded MS imaging systems that already show compact and high-quality performance in proof-of-concept devices [218]. Three-dimensional meta-optical architectures that combine multiple planar layers or folded optical paths will be essential for realizing complete imaging and spectroscopic systems within millimeter or submillimeter thicknesses. At the same time, compatibility with standard semiconductor fabrication processes remains a key enabler for large-scale deployment, as evidenced by commercial efforts in flat optics and foundry-compatible MS platforms targeting mass production [205]. These trends support a future in which hybrid plasmonic and MS photonics provides a unified design framework for planar, programmable, and computationally aware optical systems [172].

**Figure 14:**
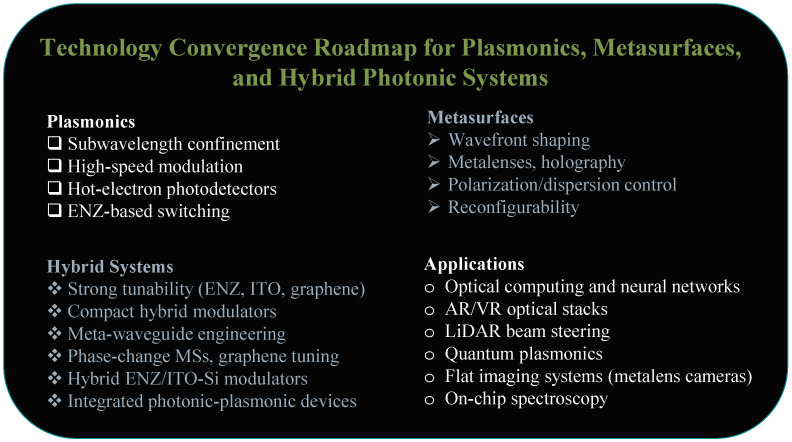
Roadmap illustrating the functional domains of plasmonics, MSs, and hybrid photonic systems, and their connection to emerging optical applications.

To synthesize these directions at the system level, Figure 14 presents a consolidated roadmap linking plasmonic, MS, and hybrid planar platforms to emerging application domains. As illustrated, MS-based imaging, sensing, and beam steering technologies are positioned for near term commercialization due to their reliance on predominantly passive functionality and strong compatibility with wafer-scale manufacturing [219,220,221]. In contrast, optical computing and highly reconfigurable photonic processors offer substantial long-term impact but face higher barriers related to system integration, programmability, power consumption, and thermal management [111,222]. This progression suggests a staged development pathway in which passive and quasi-static planar optics mature first, followed by increasingly complex active and computational photonic systems.

## 10. Concluding Remarks

The convergence of plasmonics and MSs marks a pivotal moment in the evolution of planar and interface-engineered photonic systems, reshaping long-standing assumptions about how optical hardware should be designed, fabricated, and optimized. Throughout this review, it has become evident that plasmonics provides unmatched field confinement, ultrafast light matter interaction, and the ability to engineer strong optical nonlinearities within deeply subwavelength volumes. At the same time, MSs introduce unprecedented control over phase, amplitude, polarization, and dispersion within planar interfaces measured in only hundreds of nanometers. When these two domains are considered jointly rather than independently, a new design space emerges in which optical functionalities traditionally requiring bulky components or long propagation lengths can be realized within compact, reconfigurable, and computationally aware architectures.

Central to this vision is the recognition that future photonic platforms will depend critically on interface-mediated control rather than bulk material propagation. Plasmonics enables modulators, detectors, and nanoscale resonant elements that operate at terahertz or even femtosecond speeds, while MSs offer versatile wavefront shaping, multiplexed information channels, and the ability to embed computational transformations directly into optical layers. Emerging hybrid systems demonstrate that these strengths are complementary rather than competitive. By combining plasmonic field enhancement with the low-loss routing and broadband manipulation offered by dielectric MSs, hybrid devices can surpass the performance of either platform alone. This synergy is already evident in applications ranging from ultracompact electro-optic modulators to multifunctional metalenses, coding MSs, and three-dimensional integrated optical processors.

Despite these opportunities, several challenges remain foundational to future progress. Loss in plasmonic materials continues to constrain efficiency for many applications. Broadband achromatic behavior in MSs is limited by dispersion, and large-area nanofabrication still requires breakthroughs in throughput, cost, and reproducibility. Thermal reliability, particularly in devices that rely on high field intensities or phase change materials, must also be addressed. These challenges are not barriers but catalysts for interdisciplinary innovation. Advances in alternative plasmonic materials such as transition metal nitrides and transparent conducting oxides, along with scalable fabrication strategies such as nanoimprint lithography, deep ultraviolet patterning, and multilayer stacking, are already beginning to close these gaps.

Looking ahead, the technological trajectory outlined in this review suggests a clear roadmap. Hybrid plasmonic and MS architectures are likely to form the backbone of next-generation planar optical systems that are flatter, faster, more energy efficient, and inherently programmable. MS-based beam steering, full color holography, reconfigurable optical interfaces, and metalens-based imaging stacks are rapidly transitioning from academic demonstrations to industrial adoption. Plasmonic modulators and detectors are poised to reshape high-speed communication and optical information processing. Diffractive and MS-based neural networks point toward optical processors that merge physical wave propagation with artificial intelligence. Together, these developments indicate that future planar photonic platforms will be characterized by compact footprints, multifunctional operation, and deep coupling with electronic and computational systems.

In essence, plasmonics and MSs are no longer parallel research directions but complementary pillars of a unified planar photonics paradigm. Their combination enables devices that process, sense, modulate, and compute optical information with efficiencies and form factors unattainable in traditional bulk or waveguide-dominated architectures. As materials continue to evolve and fabrication techniques mature, plasmonic and MS-based technologies will play a defining role in next-generation optical hardware, influencing fields as diverse as optical computing, augmented and virtual reality, quantum technologies, sensing, spectroscopy, and telecommunications. The vision that emerges is one of truly planar and programmable photonic systems capable of transforming both fundamental research and applied technological landscapes.

## Figures and Tables

**Table 1:** Key Characteristics of Plasmonics, MSs, and Hybrid Photonic Platforms.

Dimension	Plasmonics	Metasurfaces (MSs)	Hybrid Plasmonic–MS/Plasmonic–Dielectric Systems
Core Physical Strength	Extreme subwavelength confinement; ultrafast electron–mediated response; strong nonlinearities [74,75,76,77].	Complete phase, amplitude, and polarization control at planar interfaces; broadband functionality improving [78,79].	Combines strong confinement (plasmonics) with low-loss routing and wavefront control (dielectric/MS) [30,80,81,82].
Key Advantages	Enables nanometer-scale modulators, detectors, and quantum-scale hotspots; supports ENZ-based ultrafast switching [24,25,83].	Enables flat optics, ultrathin lenses, programmable apertures; supports multifunctionality and complex wavefront shaping [13,84,85,11].	Achieves high modulation efficiency with reduced loss; allows highly integrated and programmable photonics; enables compact optical processors [63,86,87].
Main Limitations (Current)	Ohmic (absorption) loss; short propagation lengths; heat generation; material stability constraints [88].	Fabrication scalability; limited broadband achromaticity; need for multi-layer integration for complex functions [89,90].	Coupling mismatch between dielectric and plasmonic modes; thermal management; fabrication tolerance demands [55,91,92].
Materials Outlook	Alternatives to Au/Ag: TiN (CMOS-compatible), Al (UV), Cu (low-cost), ITO (ENZ), graphene (tunable mid-IR) [50,93].	High-index dielectrics (Si, TiO_2_); hybrid designs combining metallic and dielectric scatterers; phase-change materials for reconfigurability [11,18,28,62].	ITO + Si, graphene + ITO, plasmonic inserts in Si waveguides; meta-waveguides with engineered dispersion and polarization control [94,95].
Device-Level Examples	Plasmonic modulators, nanolasers, hotspots for sensing, ultrathin detectors [96,97].	Metalenses, holographic processors, beam steering devices, MS-based neural networks [98,99].	Hybrid ENZ modulators, MS-enabled compact waveguides, reconfigurable intelligent photonic surfaces [100,101].
Performance Frontier	Femtosecond-scale modulation; deep subwavelength mode confinement; intense local fields for nonlinear processes [102].	System-level optical functionality: multi-frequency control, phase–amplitude–polarization multiplexing; flat optical architectures for cameras and LiDAR [103,104].	Best-of-both-worlds: high speed + low footprint + programmable wavefront control; pathways toward optical computing [105].
Scalability Outlook	Requires new plasmonic materials with lower loss and CMOS compatibility [106].	Moving toward mass production via nanoimprint, DUV lithography; multi-layer stacking likely [34,107].	Depends on seamless integration in Si photonics; improved thermal handling and coupling engineering [108].
Role in Future Optical Computing	Provides nonlinear activation, ultrafast switching, and strong light–matter interaction for deep photonic neural networks [57,109].	Provides spatial transformations and diffractive computing layers (e.g., MS neural networks) [110,111].	Forms complete photonic compute stacks: MS front-end for linear ops + plasmonic activation layers [112].
Role in Imaging and Sensing	Raman enhancement, nanoscale detectors, localized field hotspots [113,114].	Metalenses, reconfigurable holography, spectral filters and absorbers [115,116,117].	On-chip structured illumination, MS-controlled plasmonic sensors, integrated spectrometers [118,119].
Role in AR/VR and Compact Optics	Enhances sensing elements (photodetectors, modulators) [120,121].	Enables ultrathin imaging stacks, folded metalenses, beam combiners for headsets [11,122].	Compact, integrated emitter–MS–detector stacks for next-gen wearable optics [123].
Application Sectors Poised for Disruption	High-speed interconnects, ultrafast computing nodes, quantum sources [124,125].	Consumer imaging, LiDAR, 3D sensing, AR/VR displays, optical encryption [126,123,127].	Full-stack systems: flat cameras, photonic processors, adaptive optical networks [128].
Future Research Priorities	Low-loss materials, thermal management, quantum-plasmonic integration [46,129].	Broadband achromatic operation, dynamic tunability, large-area manufacturing [130,131,132].	Thermal engineering, mode-matching strategies, foundry-compatible fabrication [133].
Long-Term Vision	Plasmonics: acts as nanoscale “processing nodes” embedded in larger photonic circuits [134].	MSs: universal optical interfaces providing programmable wavefront control [135].	Hybrid platforms: planar, scalable, computationally aware optical systems defining the next photonic paradigm [136,137].

## Data Availability

No new data were created or analyzed in this study.
